# SAPPIRE: a Prototype Mobile Tool for Pressure Ulcer Risk Assessment

**Published:** 2014

**Authors:** Hyeoneui Kim, Heejoon Chung, Shuang Wang, Xiaoqian Jiang, Jeeyae Choi

**Affiliations:** 1 Division of Biomedical Informatics, University of California San Diego, La Jolla, CA, USA; 2 Division of Biomedical Informatics, Seoul National University, Seoul, Korea; 3 College of Nursing, University of Wisconsin, Milwaukee, Milwaukee, WI, USA

**Keywords:** Pressure ulcer risk assessment, data standardization

## Abstract

Accurate assessment and documentation of skin conditions facilitate communication among care providers and are critical to effective prevention and mitigation of pressure ulcer. We report developing a prototype mobile system called SAPPIRE (Skin Assessment for Pressure Ulcer Prevention, an Integrated Recording Environment) for an android device to assist nurses with skin assessment and documentation at bedside. SAPPIRE demonstrates (1) data documentation conforming to the relevant terminology standards, (2) data exchange using Continuity of Care Records (CCR) standard and (3) smart display of patient data relevant to risk parameters to promote accurate pressure ulcer risk assessment with the Braden scale. Challenges associated standardizing assessment data faced during this development and the approaches that SAPPIRE took to overcome them are described.

## 1. Introduction

Pressure ulcer is not uncommon, affecting more than 2.5 million people in the US each year [1]. Pressure ulcer is a major nursing liability that causes severe pain and discomfort to patients and consumes lots of healthcare resources for treatment. Hospital acquired pressure ulcer is considered a reflection of sub-optimal nursing care and its treatment expense is not reimbursed by Medicare and Medicaid anymore [2].

Accurately assessing the risk of developing pressure ulcer is the critical first step of its prevention. Nurses assess patients using standardized scales developed for pressure ulcer risk assessment and the Braden scale is the most widely used one in the US. Nurses take consideration of wide range of patient conditions to assess pressure ulcer risk using the Braden scale, which indicates this process also generates lots of data [3,4]. A means to improve standard based documentation and accessibility of the data relevant to pressure ulcer risk assessment can facilitate: (1) efficiency of care at bedside, (2) communication among care team members, and (3) comparative effectiveness analyses on various care strategies related to pressure ulcer.

A critical prerequisite to realizing these benefits is representing the data in an interoperable manner. Omnipresence of mobile technologies provides a unique opportunity to improve accessibility of the data. Documenting patient assessments and observations at the bedside conforming to standards using a mobile device then transfer them to the EHR system will increase not only documentation efficiency but also data quality. Similarly, making available of additional relevant data stored in the EHR system at the bedside through the mobile device during the assessment can facilitate the decision processes associated with the patient assessment. The data standards and practice guidelines in pressure ulcer care produced by nursing and informatics leadership groups provide workable foundations for implementing this idea. Health Level 7 developed the Pressure Ulcer Prevention Domain Analysis Model (PUP-DAM) [5] and the National Pressure Ulcer Advisory Panel (NPUAP) put forth the guidance to improve pressure ulcer prevention and care [6]. The Federal Health Information Model (FHIM) produced the Pressure Ulcer Content Model (PUCM) [7] where skin assessment data are mapped to the Logical Observation Identifiers Names and Codes (LOINC) [8] and Systematized Nomenclature of Medicine – Clinical Terms (SNOMED-CT) [9].

As the first step to realizing these benefits, we explore the feasibility of mobilizing data in the pressure ulcer assessment domain by developing a prototype mobile application for skin assessment called SAPPIRE (Skin Assessment for Pressure ulcer Prevention – Integrated Recording Environment). In this initial development, we focused on standard based data capture, documentation and transfer as well as workflow efficiency in assessing and documenting the data. Specifically, we aimed to test the adequacy of using (1) the standardized terminology systems designed to represent detailed patient observation data such as SNOMED-CT and LOINC to encode the data related to the pressure ulcer risk assessment and (2) the Continuity of Care Records (CCR) [10] to represent the data for messaging.

## 2. Methods

This pilot study was done using the data elements documented in the electronic flow sheets of the University of California, San Diego (UCSD) Medical Center. At UCSD Medical Center, Epic electronic medical record (EMR) system is in use both in the ambulatory and the inpatient settings. More than 100 modules of electronic flow sheets are implemented to meet the documentation needs of special care units. Basic data elements are overlapped among different modules of implementation. In total more than 8000 data items, which include standardized assessment scales such as the Braden scale, the Glasgow Coma Scale, etc., are included in these flow sheets.

### 2.1. Data items for the pilot project

We first identified 28 data items that are closely related to pressure ulcer risk assessment based on our related prior works [11,12]. We also referred to the HL7 PUP-DAM [5]and the NPUAP guidelines [6] and augmented the data item list with additional assessment items critical for pressure ulcer risk assessment. For this feasibility study, we selected 14 most common assessment items, 11 of which have predefined categorical values.

### 2.2. Data standardization

Data standardization involved in two parts. First, we mapped the local data items selected previously to the standardized terminology systems. Expanding to the value options, total 116 terms of data item names and values were included in this mapping. Following the FHIM-PUCM [7], we first attempted to map the data item names to LOINC and value terms to SNOMED-CT. If no appropriate mapping for an item name was found, we searched SNOMED-CT next. We searched Unified Medical Language System (UMLS) Metathesaurus [13] when we did not find a match for either an item name or a value term from the two systems. Two of the authors (HK, JC) with nursing and informatics background conducted the mapping. Second, we tested the feasibility of using CCR result template to represent the skin assessment data for messaging by mapping the data items associated with skin assessment (e.g., testing skin assessment items, patient information, provider information, and other administrative information) to the 18 sections of the CCR template (i.e., demographics, insurance information, diagnosis/problem list, alerts/allergies, medications, immunizations, social history, family history, functional status, advance directives, encounters, procedures, care plan, vital signs, laboratory results, medical equipment, providers, support persons) [10].

### 2.3. Application development

The application development was done using the android platform version 4.1.1. Main focus areas of this development were: (1) capturing skin assessment information including skin images, (2) encoding the assessment data with standardized terminology systems, and (3) messaging the data using CCR. We also intended to embed the functions that support the pressure ulcer risk assessment such as smart display of relevant patient data^(11,12)^ and the Braden scale parameter definitions.

**Figure 1:**
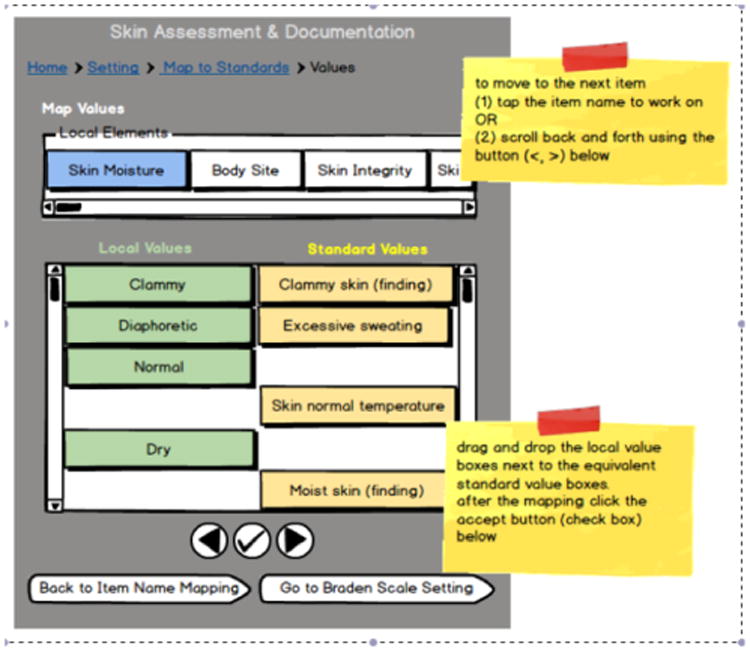
Mock-up for the value standardization screen

The team first generated a list of functions that need to be implemented in four general steps of using this application for skin assessment: (1) application set-up (importing the local data items to document and mapping them to standardized terminologies, importing patient cases to work on, and associating assessment data to the Braden scale parameters), (2) conducting skin assessment and documenting data using the application, (3) completing pressure ulcer risk assessment with the Braden scale based on the prior assessment, and (4) exporting the generated data in an interchangeable format. Once the specific functions were identified, the team produced mock up screens with detailed annotations on the system functions (Figure 1). The prototype SAPPIRE was tested with 5 patient scenarios. The functional errors and inefficiencies identified during the testing process were corrected and improved.

## 3. Results

### 3.1. Terminology mapping

LOINC provided standardized names for 11 of the 14 assessment item names. The 3 assessment items whose names were mapped to SNOMED-CT instead were *device type* (type of device: 260846005), *induration* (induration of skin: 34319007), and *bladder pattern* (urinary flow pattern: 251991007). SNOMED-CT provided exact matches to 107 of the 112 value terms. Two of the unmapped value terms *ashen* (change in color of skin to gray or ashen: C2045660) and *weeping edema* (weeping edema: C2938876) were mapped to the UMLS Metathesaurus concepts. Three value terms that were not mapped to any of the target terminology systems were *even tone* (skin), *ruddy* (skin color), and *not assessed*. The *skin temperature* item was mapped to three concepts (i.e., skin temperature, skin moisture, skin turgor) as its value list contained the three assessment areas. A *skin turgor* value tenting was mapped to two concepts: a main concept (decreased skin turgor: 425244000) and a qualifier concept (severe: 24484000).

Excluding anatomic location item and standardized assessment scales, the mapped concept unique identifiers (CUI) of the 9 assessment items and their values are presented in Table 1 along with the mapped source terminology systems. The value terms are listed under the corresponding item name terms with indentation.

### 3.2. The application

**Figure 2:**
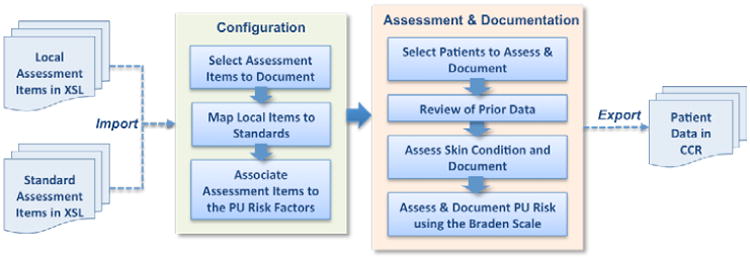
SAPPIRE workflow

**Figure 3:**
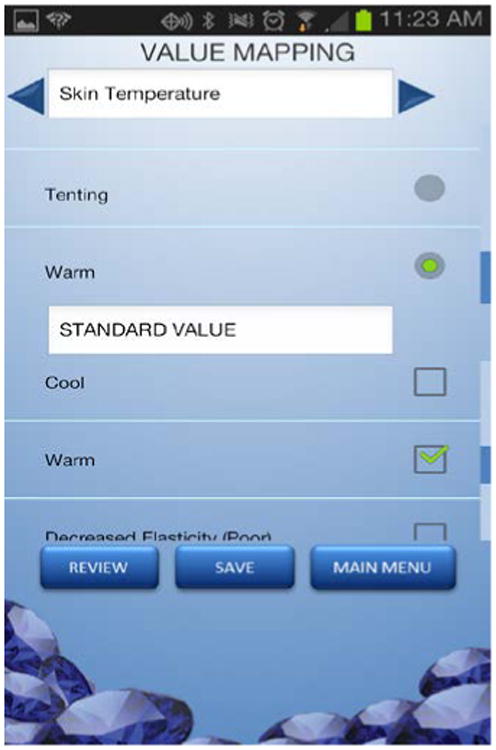
Screenshot of value mapping

The overall SAPPIRE workflow and the key functions implemented in each step are presented in Figure 2. In general, the application screens were implemented in a much simpler form than the original mock-ups in this prototype. For example, Figure 3 shows how the value mapping design in Figure 1 is implemented. The application, the user manual, and testing data sets are available for download at http://dbmi-engine.ucsd.edu/ONC/.

### 3.3. CCR representation of the skin assessment data

**Figure 4:**
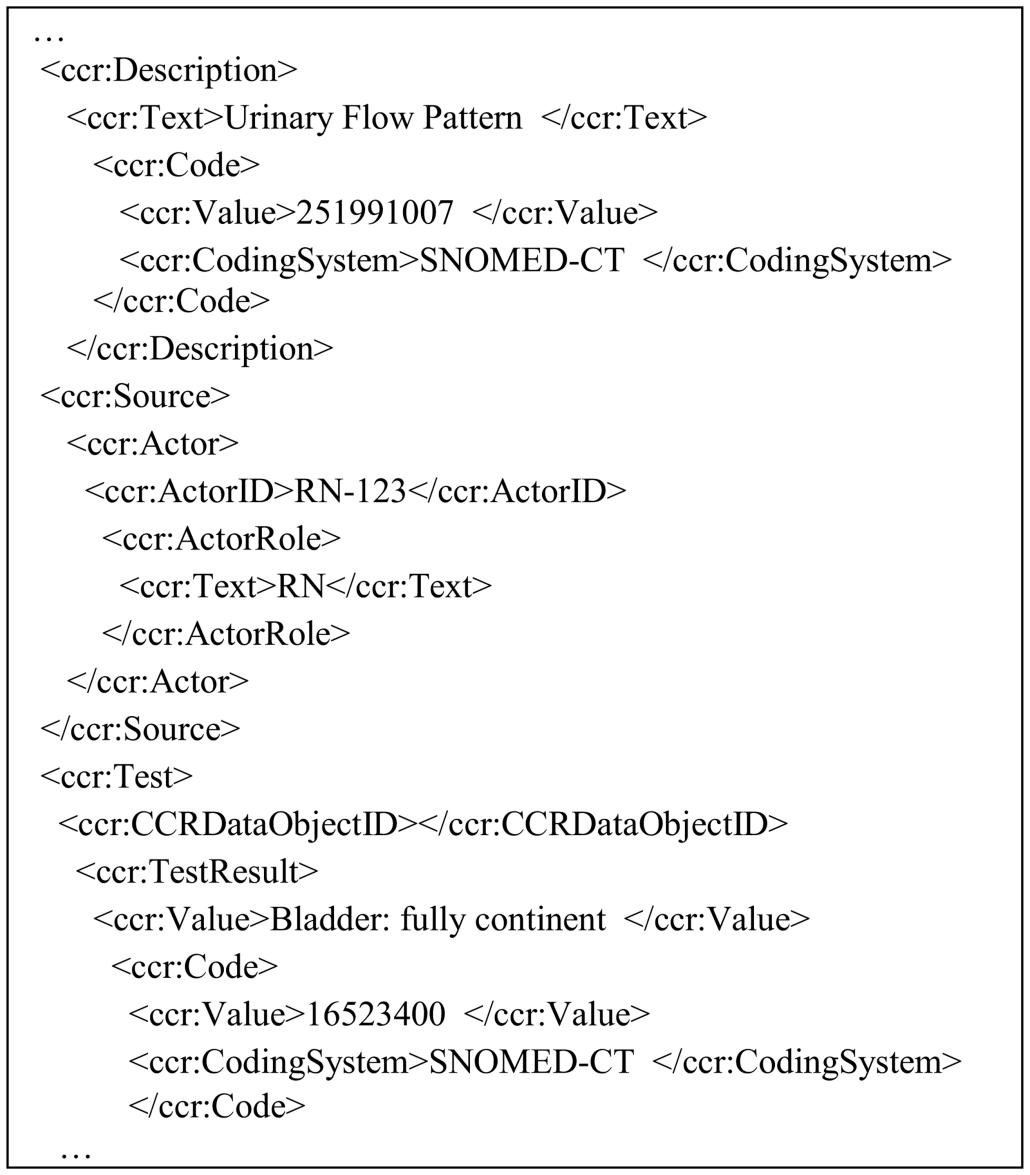
Representing urinary flow pattern data in CCR

Among the 18 sections of the CCR template we utilized demographics, diagnosis/problem list, vital signs, providers, encounters, and laboratory results sections to represent the patient scenarios and the data generated while testing SAPPIRE. Since CCR does not provide a dedicated section for daily nursing assessment observations, discrete skin assessment data involved in this study were represented using the laboratory results section. Our data were fully represented with CCR including the mapped standardized terminology codes: source terminologies were captured using the CCR elements <ccr:CodingSystem> and concepts codes were capture using <ccr:Code>. A snippet of the CCR laboratory results section that represents the data item urinary flow pattern is presented in Figure 4 as an example. The data export function of SAPPIRE converts and saves the collected patient data into a CCR file.

## 4. Discussion and Conclusion

We developed a prototype mobile application SAPPIRE for pressure ulcer risk assessment. We believe that the workflow and functionalities implemented in SAPPIRE can be easily applied to the other assessment domains. This paper described mainly the data standardization tasks. However, it is noteworthy that SAPPIRE also provides a light decision support function of displaying collected assessment data relevant to each Braden scale parameter, as defined during the configuration phase, while completing pressure ulcer risk assessment with the Braden scale.

As indicated in the PUP-DAM [5] we included various assessment data relevant to the risk factors presented in the Braden scale such as medical device (e.g., Peripherally Inserted Central Catheter), bowel and urinary patterns, and the level of consciousness (e.g., Glasgow coma scale scores) into SAPPIRE. Also, we included observations for skin blenching and indurations, which are the signs that NPUAP highly recommends to look for to detect the early stage pressure ulcer [6].

LOINC and SNOMED-CT provided exact matches to the majority of the test data items used in this pilot study. However, due to the limited scope, this mapping result by no means represents the true concept coverage of these terminology systems toward skin assessment data. We mapped the item names to SNOMED-CT when there was no match found in LOINC.

To separate the semantic roles that data item names and values play we restricted item name mapping to SNOMED-CT “observable entity” concepts unless the items take Boolean as value options, in which case item names are mapped to “clinical findings” concepts.

One of the major challenges in implementing SAPPIRE is incorporating the standards into the practical way of presenting the assessment items at the local institution. For example, the local item *skin temperature* encompasses 3 types of information such as *skin temperature on touch, skin moisture*, and *skin turgor*. In SAPPIRE, we addressed this issue by allowing users to map one data item name to multiple standardized data item names. Subsequently, the idiosyncratic value options under the skin temperature item are mapped to the standardized values that belong to the correct “type” of information.

Although the detailed assessment data are fully represented using the laboratory results section of CCR, it was somewhat counter-intuitive to map assessment and/or observation data into the laboratory result section. We suspect that having more general section name for the lab results section or adding a new section that encompasses detailed assessment and/or observation data may eliminate unnecessary confusion in using the CCR standards.

SAPPIRE demonstrates the capability of supporting standard-based capture and transfer of nursing assessment data at bedside with added decision support functions. As a proof-of-concept study, SAPPIRE was developed without a formal user requirement analysis. To be useful in a real clinical setting, SAPPIRE needs to be thoroughly evaluated and refined based on extensive user requirements, which is planned as a next step of this study.

## Figures and Tables

**Table 1:** Standardized terminology mapping of the local assessment items

Local Terms	CUI	Source	Local Terms	CUI	Source
Bowel Pattern	46016-2	LOINC	Skin Temperature	44968-6; 39129-2; 39109-4	LOINC
Fully Continent	24029004	SNOMED	Cool	427733005	SNOMED
Occasional Accident	165230005	SNOMED	Diaphoretic	52613005	SNOMED
Incontinent	72042002	SNOMED	Dry	16386004	SNOMED
Bladder Pattern	251991007	SNOMED	Edematous	95322002	SNOMED
Fully continent	16523400	SNOMED	Flaky	271767006	SNOMED
Occasional accident	165233007	SNOMED	Hot	164615004	SNOMED
Incontinent	165232002	SNOMED	Tenting	425244000; 24484000	SNOMED
Generalized Edema	44966-0	LOINC	Warm	102599008	SNOMED
+1	420829009	SNOMED	General Skin Color	39107-8	LOINC
+2	421605005	SNOMED	Flushed	248213001	SNOMED
+3	421346005	SNOMED	Jaundice	18165001	SNOMED
+4	421129002	SNOMED	Normal for ethnicity	297952003	SNOMED
Pitting	284521000	SNOMED	Pale or pallor	274643008	SNOMED
Non-Pitting	420435001	SNOMED	Pink	304230005	SNOMED
Weeping	C2938876	UMLS	Red	164424003	SNOMED
Anasarca	442433009	SNOMED	Ashen	C2045660	UMLS
Trace	260405006	SNOMED	Cyanosis	119419001	SNOMED
Generalized Skin Integrity	72300-7	LOINC	Dusky	445394005	SNOMED
Abrasion(s)	400012003	SNOMED	Ecchymosis	302227002	SNOMED
Ecchymosis	302227002	SNOMED	Even tone	Not Mapped	
Excoriation	247444006	SNOMED	Mottled	406128001	SNOMED
Laceration(s)	274165007	SNOMED	Not assessed	Not Mapped	
Lesions	95324001	SNOMED	Ruddy	Not Mapped	
Petechiae	423716004	SNOMED	Device type	260846005	SNOMED
Rash	271807003	SNOMED	Blenching Response	44971-0	LOINC
Weeping	239164002	SNOMED	Induration	34319007	SNOMED
